# Prevalence and antibiotic susceptibility of Uropathogens from cases of urinary tract infections (UTI) in Shashemene referral hospital, Ethiopia

**DOI:** 10.1186/s12879-017-2911-x

**Published:** 2018-01-10

**Authors:** Wubalem Desta Seifu, Alemayehu Desalegn Gebissa

**Affiliations:** 1 Department of Biotechnology, Wolkite University, Wolkite, Ethiopia; 2 Department of Biology, Haramaya University, Alemaya, Ethiopia

**Keywords:** Antibiotic sensitivity test, Prevalence, Urinary tract infection

## Abstract

**Background:**

Urinary tract infection (UTI) remains to be one of the most common infectious diseases diagnosed in developing countries. And a widespread use of antibiotics against uropathogens has led to the emergence of antibiotic resistant species. A laboratory based cross-sectional survey was conducted in Shashemene referral hospital to determine the prevalence and antibiotic susceptibility of uropathogens.

**Methods:**

We have collected 384 clean catch mid-stream urine samples from all suspected UTI outpatients using sterile screw capped container. The urine samples were cultured and processed for subsequent uropathogens isolation. The isolated pure cultures were grown on BiOLOG Universal Growth agar (BUG) and identified using GEN III OmniLog® Plus ID System identification protocols. The identified species were then exposed to selected antibiotics to test for their susceptibility.

**Results:**

The overall prevalence of urinary tract infection in the area was 90.1%. Most frequently isolated uropathogen in our study was *Escherichia coli* (39.3%). While, *Staphylococcus* species (20.2%), *Leuconostoc* species (11.4%), *Raoultella terrigena/Klebsiella spp./* (8.4%), *Salmonella typhimurium* (6.3%), *Dermacoccus nishinomiyaensis* (6.3%), *Citerobacter freundii* (5.2%) and *Issatchenkia orientalis/Candida krusei/* (2.7%) were the other isolates. We find that the relationship between uropathogens and some of UTI risk factors was statistically significant (*P* < 0.05). Gentamicin was the most effective drug against most of the isolates followed by chloramphenicol and nitrofurantoin. In contrast, amoxicillin, vancomycin and cephalexin were the antibiotics to which most of the isolates developed resistance.

**Conclusion:**

Urinary tract infection was highly prevalent in the study area and all uropathogens isolated developed a resistance against mostly used antibiotics.

**Electronic supplementary material:**

The online version of this article (10.1186/s12879-017-2911-x) contains supplementary material, which is available to authorized users.

## Background

Urinary tract infection remains to be one of the most common infectious diseases diagnosed in outpatients [1]. It is most often caused due to bacteria, but may also include fungal and viral infections [2]. Gram-negative bacteria cause 90% of UTI cases while gram-positive bacteria cause only 10% of the cases. The most frequent isolated uropathogen is *Escherichia coli*, accounting for 65%–90% of urinary tract infections [3, 4]. The relative frequency of uropathogens varies depending upon age, sex, catheterization, hospitalization and previous exposure of antimicrobials [5–7].

The emergence of antibiotic resistance in the management of UTIs is a serious public health issue. Particularly in the developing world where there is high level of poverty, illiteracy and poor hygienic practices, there is also high prevalence of fake and spurious drugs of questionable quality in circulation [4, 8]. The easy availability in the community without prescription and low cost make the drugs subject to abuse [9]. With regards to resistance rates in Ethiopia, a report showed that high incidence of resistance to the commonly prescribed antibiotic agents was observed in some regions [6, 10, 11].

Even though, there are few published information concerning the etiology and resistance pattern of UTIs in some hospitals of Ethiopia [6, 10–12], there was no previous study and published information on UTI in the study area. This study was conducted in order to assess the prevalence of bacterial uropathogens and their in vitro susceptibility patterns to commonly used antibiotic agents amongst outpatients with complaints of UTI in Shashemene referral hospital.

## Methods

Prior to sample collection, we obtained ethical clearance from Shashemene City Administration Health Affairs Bureau Research Ethics Review Committee (Ref. WEFB-33644/484/04) and informed consent from all research participants. Three hundred and forty eight [13] outpatients volunteered in June–December of 2016. Laboratory and questionnaire-based cross-sectional survey study was used to collect samples from the outpatients. Questionnaire was developed to assess the possible risk factors associated with UTI and register clinical profile of the volunteer. Clean catch mid-stream urine samples were collected from all UTI suspected outpatients attending Shashemene referral hospital using sterile screw capped container. Outpatients with dysuria, frequency, urgency, supra-pubic pain/tenderness and occasional hematuria were considered as possible suspects for UTI. Name, age, sex, clinical history and treatment history of the screened outpatients were recorded and the four age categories considered in this study were children (under 18), young (18–29), adults (30–45) and old (above 45) years (Additional file 1).

### Isolation of bacteria from urine samples and preservation

Urine dipstick test was done by using Multisticks of Medi-Test combi 10®SGL leukocyte esterase and nitrite [14]. The urine samples were also examined microscopically for pus cells and then inoculated on MacConkey agar and Blood agar media. Inoculated agar plates were incubated aerobically at 37 °C for 24 to 48 h. The cultured plates were examined for growth and mixed colonies on a plate were re-inoculated further on blood agar and nutrient agar medium for growth of discrete colony. Gram staining was done for all isolates as per the standard procedures and the smears were examined microscopically for their morphology and staining reactions [15].

Isolates were streaked on BUG agar for further identification using standard operation protocols for aerobic bacterial identification in GEN III OmniLog® Plus ID System of BiOLOG [16].

### Antibiotic susceptibility tests for Uropathogens

The antibiotic susceptibility test was done by the standard disk diffusion method on Mueller-Hinton agar (MHA) using commercial disks [17]. Turbidity standard protocol was followed in order to have homogenized bacterial inoculum suspension [15]. The following antibiotic discs, manufactured by Oxoid Ltd. Bashingstore Hampaire, UK were used for the disc diffusion tests: amoxicillin (AML, 30 μg), chloramphenicol (C, 30 μg), ciprofloxacin (CIP, 30 μg), gentamicin (GN, 10 μg), nalidixic acid (NA, 30 μg), nitrofurantoin (NTR, 300 μg), trimethprime-sulfamethoxazole (TMP-SMX) (SXT, 25 μg), tetracycline (TTC, 25 μg), vancomycin (VA, 30 μg), cephalexin (Ceph, 30 μg), ceftriaxone (CRO, 30 μg) [17, 18].

### Statistical methods

Our data were analyzed using SPSS for Windows, version 16.0 (SPSS, Inc., Chicago, Ill). Pearson Chi-square test was employed to test the existence of association between discrete variables. *P*-value of <0.05 was considered to indicate statistically significant differences. A binary logistic regression analysis was used to calculate odds ratio (OR); Crude Odds Ratio (COR) and Adjusted Odds Ratio (AOR) to ascertain the degree of association between risk factors and UTI.

## Results

### Prevalence of urinary tract infection among outpatients in Shashemene referral hospital

We examined a total of 384 (Table 1) outpatients with complaints of urinary tract infection in Shashemene referral hospital and found 90.1% overall prevalence of UTI in the study area (Table 2). The laboratory test results indicate that all samples 384 (100%) were positive for leukocyte esterase, while 88.5% were positive for nitrite and 11.5% were negative (Table 2). On the basis of microscopy of urine, it was found that 90.1% of the samples were positive for both pyuria and bacteriuria (Table 2). Of the total urine samples, 346 (90.1%) were positive and 38 (9.9%) were negative for the growth of different uropathogens on blood agar media (Additional file 2: Fig. S1.1c). On the other hand, 340 (88.5%) were positive and 44 (11.5%) were negative on MacConkey’s agar (Table 2; Additional file 2: Fig. S1.1b).

**Table 1:** Number of outpatients enrolled in the study and their corresponding age group

Age group	Gender		
	Female	Male	Total
<18	10 (2.6%)	2 (0.5%)	12 (3.1%)
18–29	92 (24%)	31 (8.1%)	123 (32%)
30–45	68 (17.7%)	36 (9.4%)	104 (27.1%)
>45	96 (25%)	49 (12.8%)	145 (37.1%)
Total	266 (69.3%)	118 (30.7%)	384 (100%)

**Table 2:** Characteristics of patients at time of presentation with symptoms of cystitis or pyelonephritis and their association with positivity of uropathogens in the study area

Characteristics		The frequency (%) of occurrence of clinical symptoms		The prevalence (%) of UTI		P-value	X^2^
Clinical symptoms				Positive	Negative		
	Fever	Yes	165 (43)	155 (44.8)	10 (26.3)	0.002*	9.287
		No	219 (57)	191 (55.2)	28 (73.7)		
	Dysuria	Yes	73 (19)	66 (19.1)	7 (18.4)	0.494	0.468
		No	311 (81)	280 (80.9)	31 (81.6)		
	Urgency	Yes	265 (69)	250 (72.3)	15 (39.5)	0.000*	20.69
		No	119 (31)	96 (27.7)	23 (60.5)		
	Frequency	Yes	231 (60.2)	210 (60.7)	21 (53.3)	0.010*	6.597
		No	153 (39.8)	136 (39.3)	17 (44.7)		
	Flank pain	Yes	220 (57.3)	200 (57.8)	20 (52.6)	0.066	3.385
		No	164 (42.7)	146 (42.2)	18 (47.4)		
	Supra-pubic pain	Yes	266 (69.3)	262 (75.7)	4 (10.5)	0.000*	39.917
		No	118 (30.7)	84 (24.3)	34 (89.5)		
	Age categories (year)	No of positive (%)					
		Female	Male	Total			
Age	<18	9 (2.34)	1 (0.26)	10 (2.6)			
	18–29	86 (22.4)	29 (7.55)	115 (29.94)			
	30–45	58 (15.1)	29 (7.55)	87 (22.65)			
	>45	90 (23.43)	44 (11.45)	134 (34.89)			
	Total	243 (63.3)	103 (26.8)	346 (90.1)			
Urinalysis and urine microscopy		No of positive	No of negative				
	Leucocyte esterase	384 (100)	–				
	Nitrite	340 (88.5)	44 (11.5)				
	Bacteruria	346 (90.1)	38 (9.9)				
	Pyuria	346 (90.1)	38 (9.9)				
	MacConkey Agar	346 (90.1)	38 (9.9)				
	Blood Agar	340 (88.5)	44 (11.5)				

*Statistically significant at P < 0.05

From the total patients with UTI compliant, 134 (34.89%) were in the old age group while 115 (29.94%) were in the young age group (Table **2**).

### Clinical symptoms associated with urinary tract infection

Clinical symptoms of UTI are the result of a complex series of host pathogen interactions that could lead to bacterial invasion and persistence and ultimately to disease [19]. In this study, clinical symptoms were used in the diagnosis to determine the course of infections.

Data of clinical symptoms and their associations with UTI of study subjects is shown in Table 2. Of the total outpatients, 155 (44.8%), 66 (19.1%), 250 (72.3%), 210 (60.7), 200 (57.8) and 262 (75.5) of them showed fever, dysuria, urgency, frequency, flank pain and supra-pubic pain respectively. Statistical analysis revealed that there is significant relation between the majority of clinical symptoms (fever, urgency, frequency, suprapubic pain) and UTI (*P* < 0.005).

### The prevalence of Uropathogens from urine samples of UTI positive patients

The relative prevalence of uropathogens isolated from mid-stream urine samples is shown in Table 3. Totally, 429 isolates of ten different kinds of uropathogens were identified from the urine samples. Of these, 417 (97.2%) belonged to bacteria while the rest, 12 (2.8%) were fungi.

**Table 3:** Prevalence of uropathogens among positive patients by sex, place of residence and age group

Identified uropathogens	Midstream urine sample								
	Sex		Residence		Age group				
	Female (%)	Male (%)	Urban (%)	Rural (%)	<18 (%)	18–29 (%)	30–45 (%)	>45 (%)	Total (%)
Gram-negative uropathogens									
*Escherichia coli*	121 (39.3)	48 (39.7)	65 (40.9)	104 (38.5)	3 (37.5)	27 (29.0)	127 (42.5)	12 (41.4)	169 (39.4)
*Raoultella terrigena*	25 (8.1)	11 (9.1)	17 (10.7)	19 (7.0)	1 (12.5)	11 (11.8)	24 (8.0)	0	36 (8.38)
*Salmonella Typhimurium*	16 (5.2)	11 (9.1)	11 (6.9)	16 (5.9)	1 (12.5)	5 (5.4)	19 (6.4)	2 (6.9)	27 (6.29)
*Citerobacter freundii*	17 (5.5)	5 (4.1)	3 (1.9)	19 (7.0)	–	3 (3.2)	17 (5.7)	2 (6.9)	22 (5.12)
Gram-positive uropathogens									
*Staphylococcus intermedius*	21 (6.8)	14 (11.6)	15 (9.4)	20 (7.4)	–	8 (8.6)	24 (8.0)	3 (10.3)	35 (8.15)
*Staphylococcus epidermidis*	38 (12.3)	14 (11.6)	18 (11.3)	34 (12.6)	1 (12.5)	18 (19.4)	31 (10.4)	2 (6.9)	52 (12.12)
*Leuconostoc citreum*	22 (7.1)	6 (4.9)	11 (6.9)	17 (6.3)	1 (12.5)	8 (8.6)	18 (6.0)	1 (3.4)	28 (6.52)
*Dermacoccus nishinomiyaensis*	22 (7.1)	5 (4.1)	7 (4.4)	20 (7.4)	1 (12.5)	5 (5.4)	20 (6.7)	1 (3.4)	27 (6.29)
*Leuconostoc mesenteroides*	14 (4.5)	7 (5.8)	7 (4.4)	14 (5.2)	–	4 (4.3)	16 (5.4)	1 (3.4)	21 (4.89)
Fungus									
*Issatchenkia orientalis*	12 (3.9)	0	5 (3.1)	7 (2.3)	–	4 (4.3)	3 (1.0)	5 (17.2)	12 (2.79)
Total	308 (71.7)	121 (28.3)	159 (37.1)	270 (62.9)	8 (1.86)	93 (21.67)	299 (69.69)	29 (6.75)	429 (100)

**Fig. 1:**
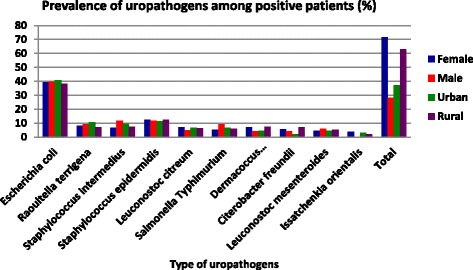
Sex and place of residence based prevalence of uropathogens

The most frequently isolated microbial species was *Escherichia coli* (39.3%). *Staphylococcus* species (20.2%), *Leuconostoc* species (11.4%), *Raoultella terrigena/Klebsiella spp.* (8.4%), *Salmonella typhimurium* (6.3%), *Dermacoccus nishinomiyaensis* (6.3%), *Citerobacter freundii* (5.2%) and *Issatchenkia orientalis/Candida krusei/* (2.7%) were the other isolated microbes (Fig. 1).

### Prevalence of uropathogens among UTI positive patients by sex

Our result shows, of the total positive patients for uropathogens, while 71.7% were female, 28.3% were male (Table 3). Statistical analysis revealed that there was a significant relationship between sex and the prevalence of uropathogens *P* = 0.041, X^2^ = 4.192 and AOR = 2.396 (Table 4).

**Table 4:** Multivariate logistic regression of risk factors for the prevalence of UTI in male and female patients

Association between risk factors and UTI							
Risk factors	The frequency (%) of occurrence of risk factors		The prevalence (%) of UTI		*P*-value	X^2^	AOR (95% CI)
			Negative	Positive			
Catheter use	Yes	39(10.2)	3(7.9)	36(10.4)	0.628	0.235	0.738(0.216–2.555)
	No	345(89.8)	35(92.1)	310(89.6)			1
Severe underlying illness	Yes	47(12.2)	2(5.3)	45(13.0)	0.183	1.771	0.372(0.086–1.597)
	No	337(87.8)	36(94.7)	301(87.0)			
Improper storage	Yes	247(64.3)	10(26.3)	237(68.5)	0.000*	21.882	0.164(0.077–0.350)
	No	137(35.7)	28(73.7)	109(31.5)			
Place of residence	Rural	223(58.1)	29(76.3)	214(61.8)	0.000*	17.316	5.224(2.398–11.381)
	Urban	161(41.9)	9(23.7)	132(38.2)			1
Age	Child	12(3.1)	1(2.6)	11(3.2)	0.900	0.016	1.158(0.11–11.491)
	Young	123(32)	13(34.2)	110(31.8)	0.302	1.066	1.580(0.663–3.767)
	Adult	104(27.1)	6(15.8)	98(28.3)	0.022*	5.235	3.404(1.192–9.720)
	Old	145(37.8)	18(47.4)	127(36.7)			1
Sex	Female	266(69.3)	23(60.5)	243(70.2)	0.041*	4.192	2.396(1.038–5.531)
	Male	118(30.7)	15(39.5)	103(29.8)			1

N.B.*significant at p < 0.05, Numbers in bracket indicates percentages, *AOR* Adjusted Odds Ratio, *CI* Confidence Interval and 1 = Reference category

### Prevalence of uropathogens among UTI positive patients by place of residence

For all uropathogens isolate, the highest prevalence was observed in patients from the rural area (62.9%) than patients from the urban (37.1%) (Table 3). Statistical analysis revealed that there is significant relation between place of residence and UTI causing microorganisms (X^2^ = 13.089, *P* = 0.000, COR = 4.648) (Table 5).

**Table 5:** Univariate logistic regression of risk factors for the prevalence of UTI in male and female patients

Association between risk factors and UTI							
Risk factors	The frequency (%) of occurrence of risk factors		The prevalence (%) of UTI		*P*-value	X^2^	COR (95% CI)
			Positive	Negative			
Catheter use	Yes	39(10.2)	3(7.9)	36(10.4)	0.348	0.881	0.507(0.122–2.096)
	No	345(89.8)	35(92.1)	310(89.6)			1
Severe underlying illness	Yes	47(12.2)	2(5.3)	45(13.0)	0.082	3.033	0.240(0.048–1.196)
	No	337(87.8)	36(94.7)	301(87.0)			1
Improper storage	Yes	247(64.3)	10(26.3)	237(68.5)	0.000*	23.691	0.121(0.052–0.284)
	No	137(35.7)	28(73.7)	109(31.5)			1
Place of residence	Rural	223(58.1)	29(76.3)	214(61.8)	0.000*	13.089	4.648(2.022–10.683)
	Urban	161(41.9)	9(23.7)	132(38.2)			1
Age	Child	12(3.1)	11(3.2)	1(2.6)	0.737	0.113	1.436(0.174–11.883)
	Young	123(32)	110(31.8)	13(34.2)	0.716	0.132	1.152(0.537–2.468)
	Adult	104(27.1)	98(28.3)	6(15.8)	0.085	2.97	2.332(0.891–6.106)
	Old	145(37.8)	127(36.7)	18(47.4)			1
Sex	Female	266(69.3)	243(70.2)	23(60.5)	0.206	1.60	1.569(0.781–3.153)
	Male	118(30.7)	103(29.8)	15(39.5)			1

N.B.*significant at p < 0.05, Numbers in bracket indicates percentages, *AOR* Crude Odds Ratio, *CI* Confidence Interval and 1 = Reference category

### Prevalence of uropathogens among UTI positive patients by age group

The highest prevalence of microbial isolates was observed in adult age group (69.69%) followed by the young (21.67%) (Table 3) and it was statistically significant *P* = 0.022, X^2^ = 5.235 and AOR = 3.404 (Table 4). This might be due to active sexuality of the age group [20, 21]. However, it will require further investigations to validate.

### Risk factors to urinary tract infection

We have considered various risk factors that might play a role in escalating UTI such as sex, age, spermicide or diaphragm use, catheter use, severe underlying illness, genital hygiene, frequent sex and improper urine storage.

Logistic regression analysis was used to calculate odds ratio (OR) to ascertain the degree of association between these risk factors and UTI. Both urine storage and place of residence had positive and statistically significant relationships with UTI (X^2^ = 23.691, *P* = 0.000 COR = 0.121 and X^2^ = 13.089, P = 0.000 COR = 4.648, respectively) (Table 5).

Among risk factors pertinent to females, statistical analysis revealed that there was no significant relationship between use of diaphragm and the prevalence of UTI (*P* > 0.05) (Additional file 3: Table S1a and b). This might be due to the fact that most females were not using diaphragm.

### Antibiotic susceptibility test

In-vitro antibiotic susceptibility tests were done on a total of 30 isolates using a standard method of agar disk diffusion technique following the National Committee for Clinical Laboratory Standards. Eleven antibiotic agents were used for the test (Amoxicillin, Chloramphenicol, Ciprofloxacin, Gentamicin, Nalidixic acid, Nitrofurantoin, Trimethprime-Sulfamethoxazole (TMP-SMX), Tetracycline, Vancomycin, and Cephalexin, Ceftriaxone (Additional file 2: Fig. S1.6a–c).

As it can be seen from Additional file 4: Table S2, 93.3%, of the isolates were sensitive to gentamicin. Similarly, 60%, 60%, 56.6%, 46.6%, 40%, 33.3%, of the isolates were sensitive to chloramphenicol, nitrofurantoin, ciprofloxacin, Trimethprime-Sulfamethoxazole (TMP-SMX), ceftriaxone and nalidixic acid, respectively. Moreover, 10–20% of the isolates were sensitive to vancomycin, tetracycline and cephalexin. On the contrary, none of the isolates showed sensitivity to amoxicillin (96.6%) followed by vancomycin (80%) and cephalexin (70%). Likewise, 40–56.6% of the isolates showed resistance to nitrofurantoin, ceftriaxone, nalidixic acid and tetracycline.

## Discussion

We have shown that the overall prevalence of UTI was 90.1%. In accordance with [22, 23], the prevalence of UTI is higher in females (63.3%) than males (26.8%) (Table 2). This might be due to the anatomical differences of urogenital organs between the two sexes [24, 25]. Prevalence difference has been also observed among various age groups. This difference suggests that age is one risk factor associated with UTI. The high incidence of UTI amongst the old age group could be due to genito-urinary atrophy and vaginal prolapse after menopause in female which in turn increases the risk of bacteriuria by increasing vaginal pH and decreasing vaginal *Lactobacillus* thereby allowing gram-negative bacteria to grow and act as uropathogens [26]. Moreover, it was indicated in another study [21, 27] that UTI is the most common infection in elderly populations. The high prevalence recorded amongst young age group could be due to increased sexual activity in the age group [26].

Fever, dysuria, urgency, frequency, flank pain and suprapubic pain were the observed clinical symptoms in our study and is comparable with report of [28]. In contrast, dysuria and flank pain were symptoms statistically not significant. Even though statistically not significant, flank pain was the symptom in which positive cases were noted in 200 (57.8%) patients, next to supra-pubic pain and urgency.

Bacterial species were the more prevalent uropathogens compared to other groups of microbes. This result is in accordance with that reported by [2] which indicated that among different microorganisms causing UTIs, bacteria accounts for more than 95% and the rest may also include fungal and viral infections. Among the isolates, gram-negative bacteria, gram-positive bacteria and fungi constituted 59.2%, 38% and 2.7%, respectively. The highest prevalence of gram-negative bacteria in this study is in agreement with that reported by [29, 30].

Moreover, the prevalence of *E. coli* (39.3%) in the current study is comparable with that reported from Nigeria, Zaria by [31], but higher than the reports of [32] from Brazil, [18] from Pakistan, and [33] from Mekele hospital, Ethiopia.

The prevalence of *Staphylococcus epidermidis* 12.1% in our study is also comparable with the study reported by [34] which was 13%. Similarly, the prevalence of other non–*E.coli* aerobic gram-negative rods is comparable with the study reported by [35], which was generally ranging from 5 to 10%.

*Dermacoccus nishinomiyaensis (Micrococcus nishinomiyaensis)* prevalence is 6.3%. According to the study conducted by [36], it was reported that *Dermacoccus nishinomiyaensis* is prevalent in urinary tract during microbial urethral stent colonization (MUSC). In similar study conducted by [37], urethral stents inserted during urinary tract infection were more frequently colonized (59%) by urophatogens compared to those placed in sterile urine (26%). Female sex and continuous stenting were significant risk factors for MUSC.

In agreement with [38], our study also showed that there was mixed bacterial species infection per a patient. Studies reported that mixed infections (poly-microbial) are more likely to occur in patients with underlying disorders that interfere with free urine flow. Moreover, it is frequent in those with indwelling catheter. The similarities and differences in the type and distribution of uropathogens may result from different environmental conditions and the prevailing practices in each country and region.

Furthermore, we assessed the relationship between various risk factors and UTI. Sex was one of the considered factors and the result indicated that UTI prevalence was higher in females than males for each isolate. Previously, [25, 39, 40] have shown that incidence of UTI was found to be higher in females than in males. This is probably due to multiple factors contributing to the problems among females. The first possible reason would be the anatomical feature of the female urethra, which is much shorter than males’ urethra. The Shortness of the urethra, allow the pathogens easy access to the bladder during sexual intercourse. This in turn results in increased bacterial counts in the bladder after intercourse [41, 42].

Statistical analysis showed that patients who were holding urine in their bladder for a long period of time had more probability of having UTI than those who were not holding. In a study done on risk factors of UTI in Pakistan, improper holding of urine in bladder was found to be one of the main causes of urinary tract infection, which produces a favorable environment for the growth of urinary tract pathogens [26, 43].

Among the risk factors of UTI pertinent to females, active sexuality/frequent sex/ has a positive and statistically significant relationship with the prevalence of UTI (Additional file 3: Table S1a). Statistical analysis revealed that there was a significant difference between the prevalence of UTI in patients who were practicing frequent sexual activity and those who were not (Additional file 3: Table S1a and b). This indicates that those patients who were practicing frequent sexual activity would have more probability of having UTI than those who were not. This is consistent with the findings reported by many authors. They showed the incidence of UTI is higher in sexually active females causing 75–90% of bladder infections, [21, 44, 45].

Similarly, keeping genital hygiene has a positive and statistically significant relationship with the prevalence of UTI. Statistical analysis done using both univariate and multivariate logistic regression revealed that there was a significant difference in the prevalence of the UTI between females keeping their genital hygiene and those who were not (Additional file 3: Table S1a and b). This indicates that those patients who were not keeping their genital hygiene had more probability of contracting UTI than those who were keeping their genital hygiene. This could be attributed to multiple factors probably contributing to the increasing problem of infection among these females. One of such factors was most of the female patients were from rural areas, and they have poor hygienic practices. Poor hygienic practice results in direct fecal contamination of urinary tract from the anus in females. Consequently it provides easier access to the pathogens overgrowth and ascent to bladder [41]**.**

The results also revealed that among eleven antibiotics used for susceptibility test, gentamicin was the most effective antibiotics 93.3% followed by chloramphenicol and nitrofurantoin. This might be due to the fact that gentamicin is offered in injection form and its unavailability in tablet form in the community, minimized the chance to abuse (Unpublished data).

We have shown there was multiple antibiotic resistances on many of the identified species. Thus*, E. coli, L. cetreum* and *S. typhimurium* were members resistant to more than five antibiotics while the rest of the isolates were resistant to three to five antibiotics. The development of higher resistance against the above-mentioned antimicrobials could be due to repeated use or prolonged exposure of uropathogens to the antibiotics [46]. Repeated use of antibiotics can damage peri-urethral flora, allowing uropathogens to colonize and subsequently infect the urinary tract. Hence, leaving clinicians with very few choices of drugs for the treatment of UTI. Moreover, this condition enables bacteria to exchange their genetic material through horizontal gene transfer resulting in resistant gene that confer resistance to a particular antibiotic [47].

## Conclusions

Urinary tract infection is the most common problem throughout the world, particularly in developing countries. In addition, emergence of bacterial strains resistant to commonly used antibiotic agents is widespread phenomenon all over the world. From the results of our study, we concluded that, UTI is prevalent in the study area and the most frequently isolated uropathogen was *E. coli* followed by *Staphylococcus spp.* In addition, *Leuconostoc* species, *Raoultella terrigena (Klebsiella spp)*, *Salmonella typhimurium*, *Dermacoccus nishinomiyaensis*, *Citerobacter freundii* and *Issatchenkia orientalis* were isolated. Female sex, poor hygienic practice of the rural residents, improper urine storage, frequent sex and lack of genital hygiene, were the major risk factors for the high prevalence of UTI. Gentamycin was the most effective antibiotic for the area followed by chloramphenicol and nitrofurantoin. In contrast, amoxicillin, vancomycin and cephalexin were the drugs to which the isolates developed resistance. Generally, as there was no previous study and published information on UTI in the study area, this study has provided baseline data on the prevalence, drug sensitivity, and some potential risk factors of UTI and is, therefore, of clinical and epidemiological significance.

## Additional files

Additional file 1: Questionnaire. (DOCX 102 kb)

Additional file 2: **Figures S1.** Different pictures of laboratory processes. The picture indicates the detail of processes followed in the study including outpatients interview, bacterial inoculation and incubation and further analyses. (DOC 1081 kb)

Additional file 3: **Tables S1.** Univariate and Multivariate logistic regression of risk factors pertinent to females for the prevalence of UTI. Univariate and multivariate logistic regression were used to correlate risk factors and prevalence of UTI. Except diaphragm use, the rest risk factors have significant association with prevalence of UTI in females. (XLSX 44 kb)

Additional file 4: **Table S2.** The proportion of sensitive, intermediate and resistant bacterial isolates to eleven different antibiotics. The table indicates the sensitivity of isolates to various antibiotics used in the study. (XLSX 41 kb)

## Abbreviations

BUG: BiOLOG Universal Growth medium
DM: Diabetes mellitus
MUSC: Microbial Urethral Stent Colonization
TMP-SMX: Trimethprime-Sulfamethoxazole
UTI: Urinary Tract Infection
