# Supplementary material for: Prevalence and antibiotic susceptibility of Uropathogens from cases of urinary tract infections (UTI) in Shashemene referral hospital, Ethiopia
Source: BMC Infect Dis. 2018 Jan 10;18:30. doi: 10.1186/s12879-017-2911-x (PMC5763535; doi:10.1186/s12879-017-2911-x)
Supplement: Supplementary file 1 — Questionnaire. (DOCX 102 kb) [file 12879_2017_2911_MOESM1_ESM.docx]

## Questionnaire

**Questionnaire for assessment of risk factors associated with urinary tract infection, clinical profile and laboratory data of outpatients at Shashemene Referral Hospital, West Arsi Zone, Oromia, Ethiopia.**

**I. Patient Identification**

1. Serial No________________________
2. Age:______ Sex: M F
3. Residence: Urban Rural
4. Marital status **:**

Single Married Divorced Widowed

1. Religion: Christian Muslim
2. Patient type Outpatient
3. Do you use spermicides /diaphram during sexual intercourse? (for female) Yes No
4. Did you use catheter? Yes No
5. Did you encounter severe underlying illness? Yes No
6. Do you make sex frequently? (For female)? Yes No
7. Do you keep your genital organs hygiene & wear dry under wear? Yes No
8. Do you store urine in bladder for long time? Yes No

**II. Clinical simptoms**

YES NO

1. Fever ____ ____

2. Dysuria ____ ____

3. Urgency ____ ____

4. Frequency ____ ____

5. Flank pain ____ ____

6. Suprapubic pain ____ ____

**III. Laboratory Data**

1. Date of urine collection _______________________________

2. Type of specimen: Mid-stream urine sample

3. Culturing and isolation of bacteria: Significant bacteruria: Yes No

4. Identification steps for isolated colonies:

Inoculation of isolated colonies on BUG agar for further identification of strains in OmniLog _________

1. Delivering of a pure culture on Biolog media ___________________

2. Inoculating MicroPlates and loading into the OmniLog______________

Name of the bacteria identified__________________________

**IV. Antimicrobial susceptibility testing S I R**

1. Ampicillin (AMP, 10μg) _______ ______ _______

2. Amoxicillin (AML, 30μg) _______ ______ _______

3. Chloramphenicol (C, 30μg) _______ ______ _______

4. Ciprofloxacin (CRO, 30μg) _______ ______ _______

5. Gentamicin (CN, 10μg) _______ ______ _______

6. Methicillin (MET, 5μg) _______ ______ _______

7. Nalidixic acid (NA, 30μg) _______ ______ _______

8. Nitrofurantoin (F, 300μg) _______ ______ _______

9. Norfloxacin (NOR, 5μg) _______ ______ _______

10. TMP-SMX (SXT, 25μg) _______ ______ _______

11. Vancomycin (VA, 30μg) _______ ______ _______

**V. Comments________________________________________________________**

__________________________________________________________
