# Supplementary material for: Prevalence and antibiotic susceptibility of Uropathogens from cases of urinary tract infections (UTI) in Shashemene referral hospital, Ethiopia
Source: BMC Infect Dis. 2018 Jan 10;18:30. doi: 10.1186/s12879-017-2911-x (PMC5763535; doi:10.1186/s12879-017-2911-x)
Supplement: Supplementary file 2 — Figures S1. Different pictures of laboratory processes. The picture indicates the detail of processes followed in the study including outpatients interview, bacterial inoculation and incubation and further analyses. (DOC 1081 kb) [file 12879_2017_2911_MOESM2_ESM.doc]

Additional file 2: Figure S1. Different pictures of laboratory processes

1.1. Inoculation and growth of bacteria on different media


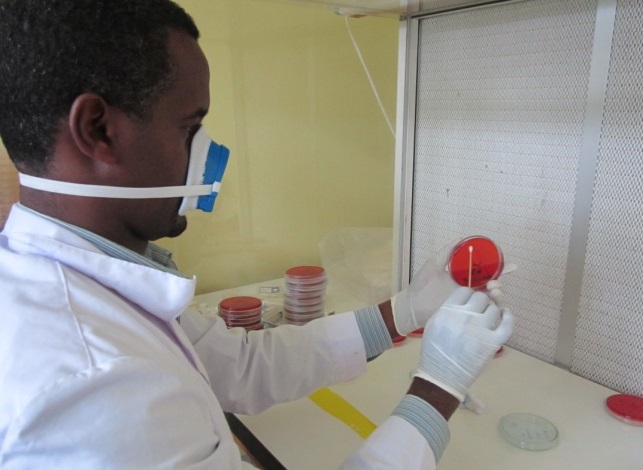

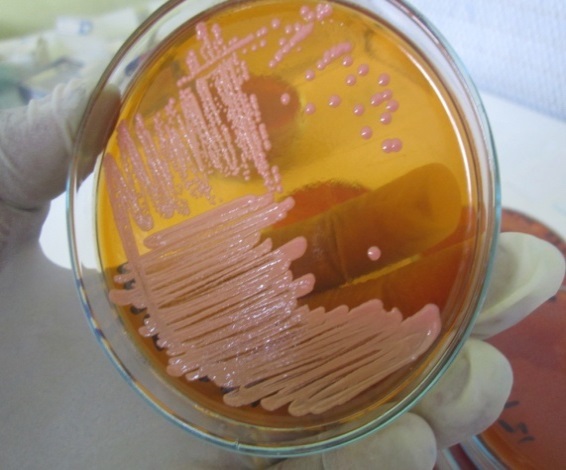


1. Inoculation b) Growth on MacConkey agar


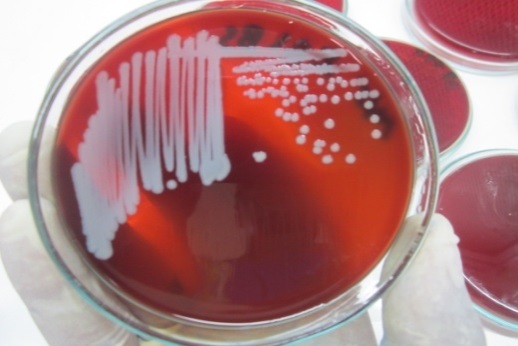

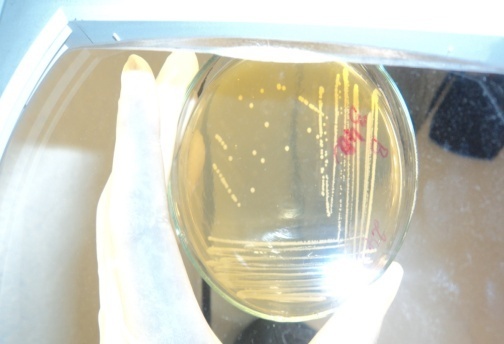


1. Growth on blood agar d) Growth on BUG agar

1.2. Gram staining and microscopy


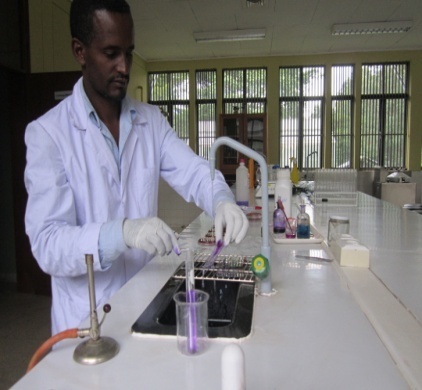

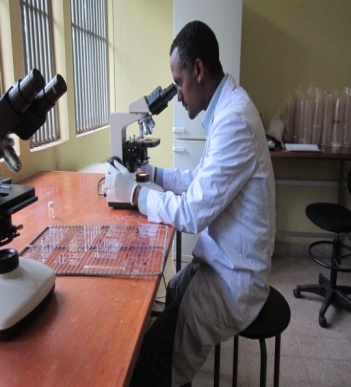

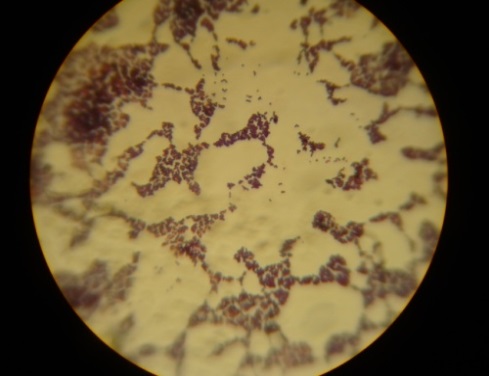


1. Staining b) microscopy c) microscopic view of bacteria


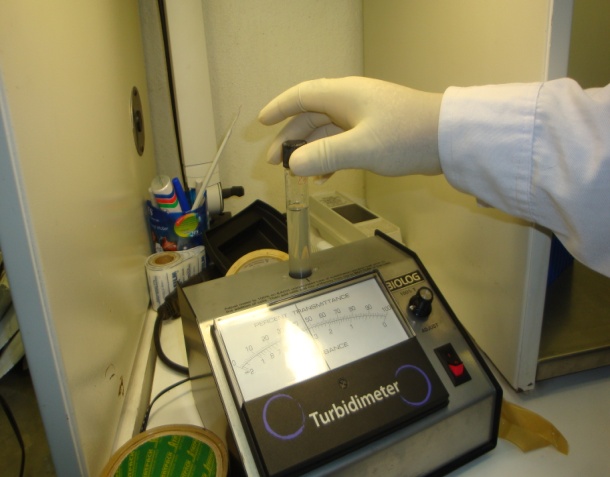

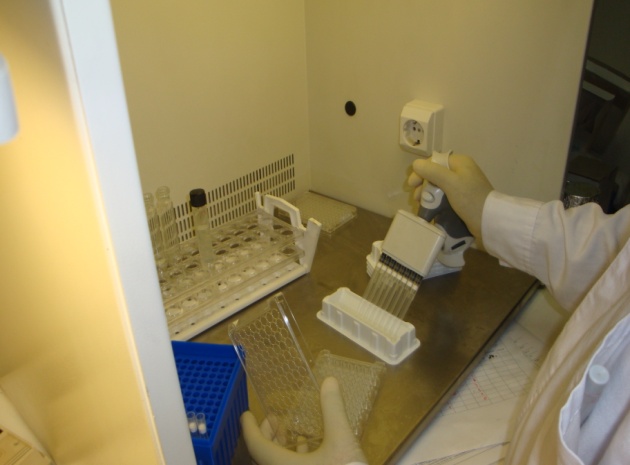


1.3. Turbidity measurement 1.4. Dispensing prepared inocula into

MicroPlate for OmniLog


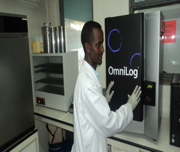

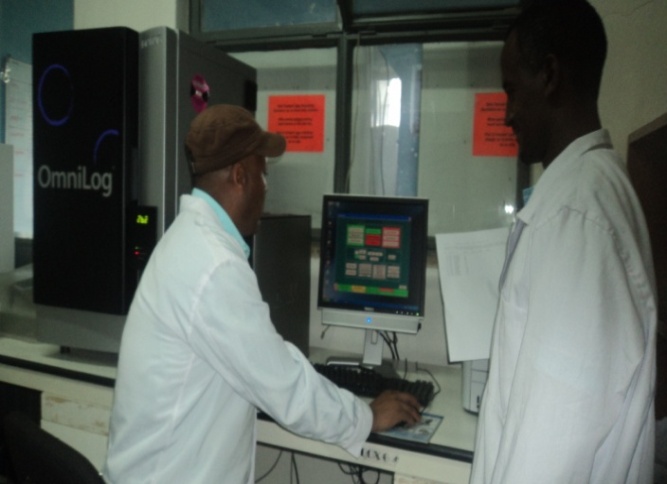


1.5. OmniLog machine and bacteria identification process

1.6. Antibiotic susceptibility test


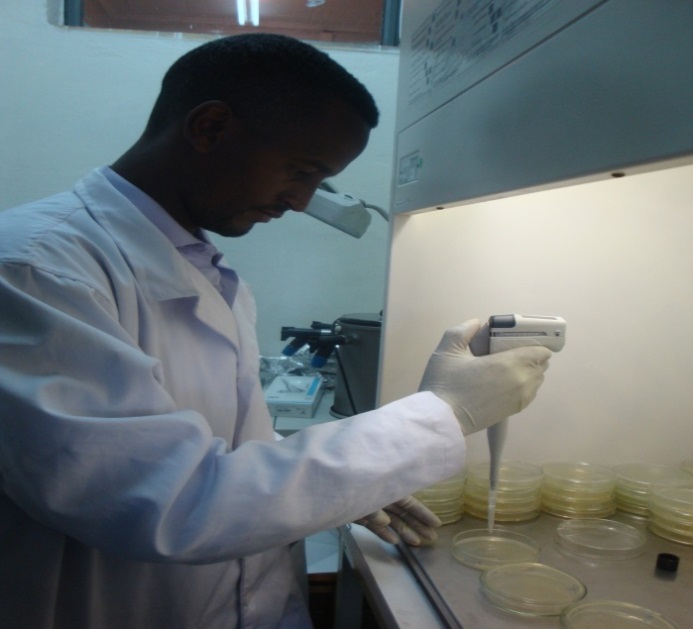


1. Transferring bacterial suspension in to Mueller-Hinton agar


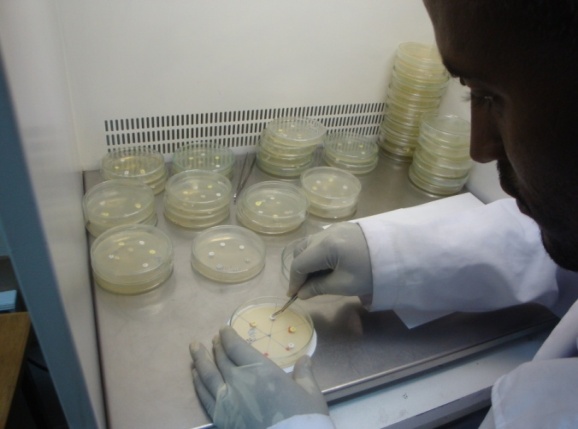

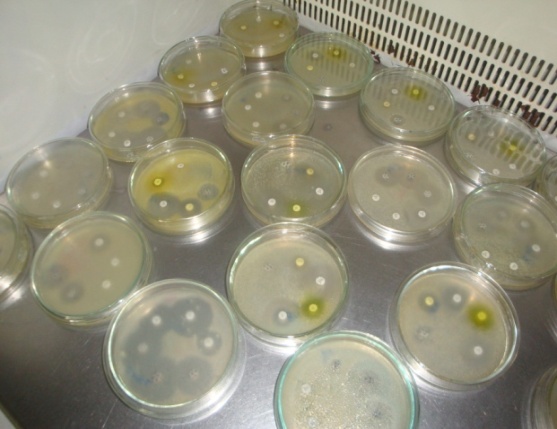


1. Gently pressing down of discs on to c) Diameters of the zone of inhibition

Mueller-Hinton agar surface around the discs
